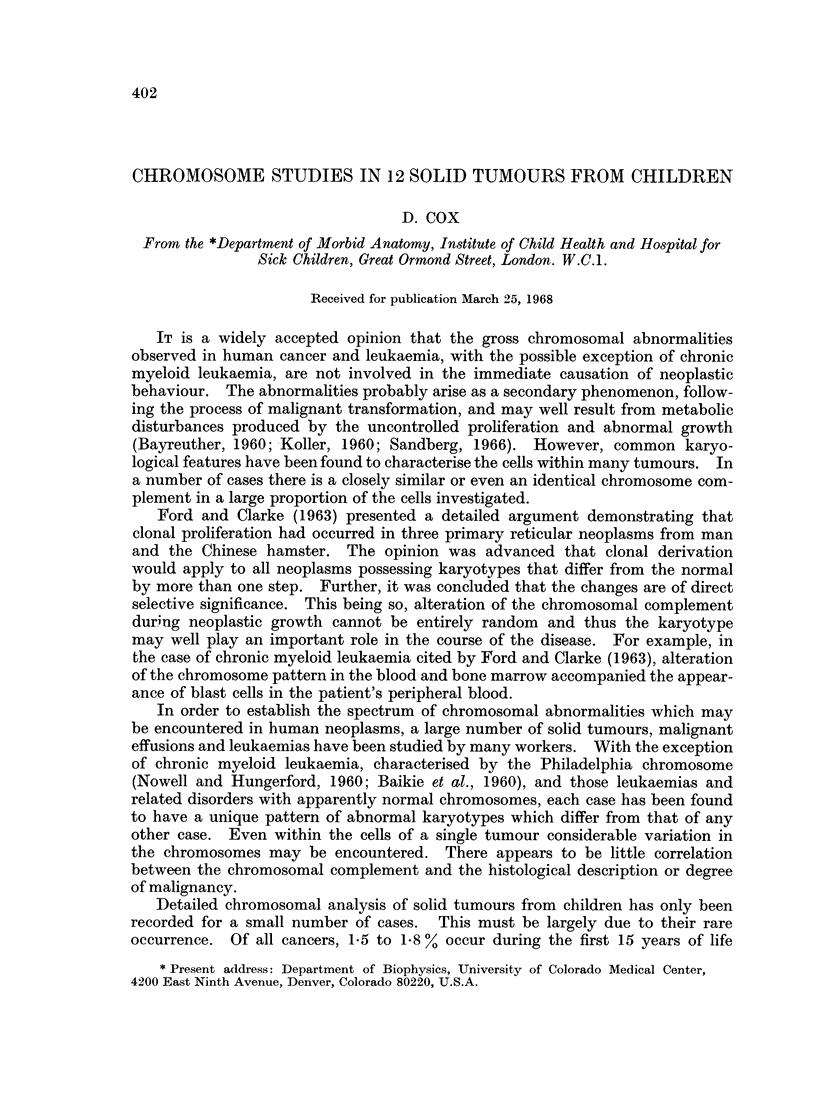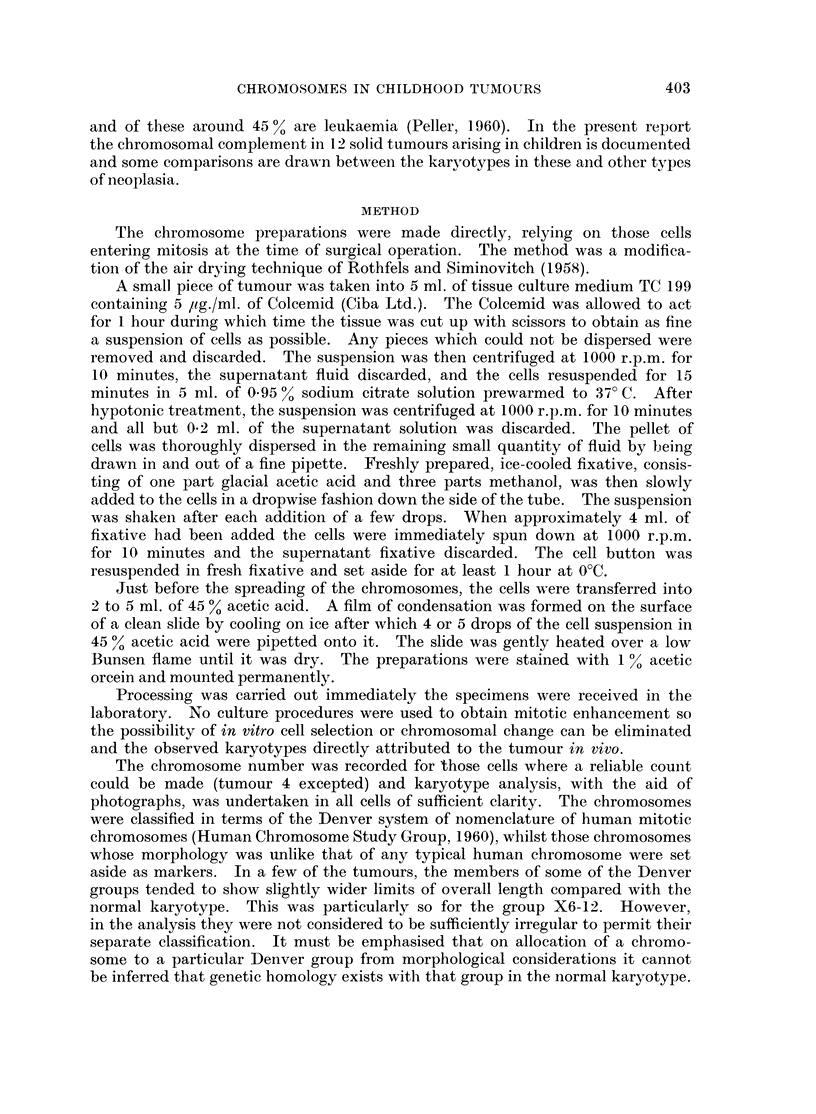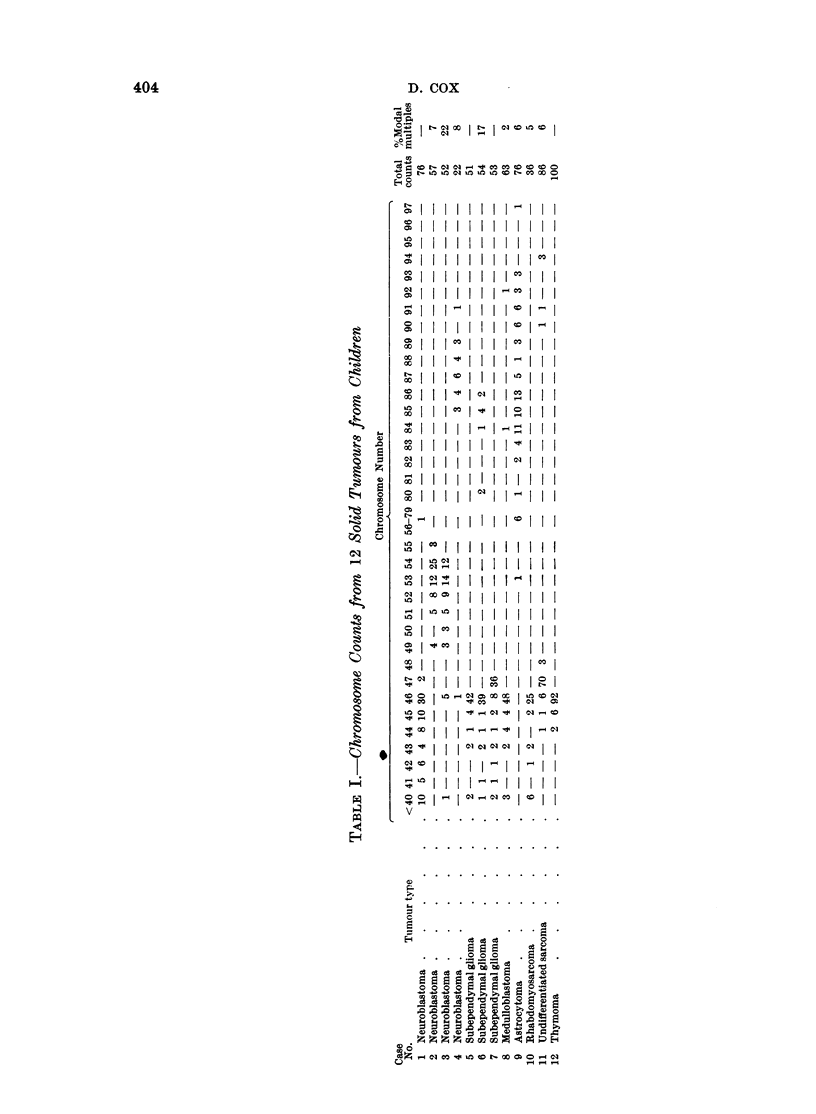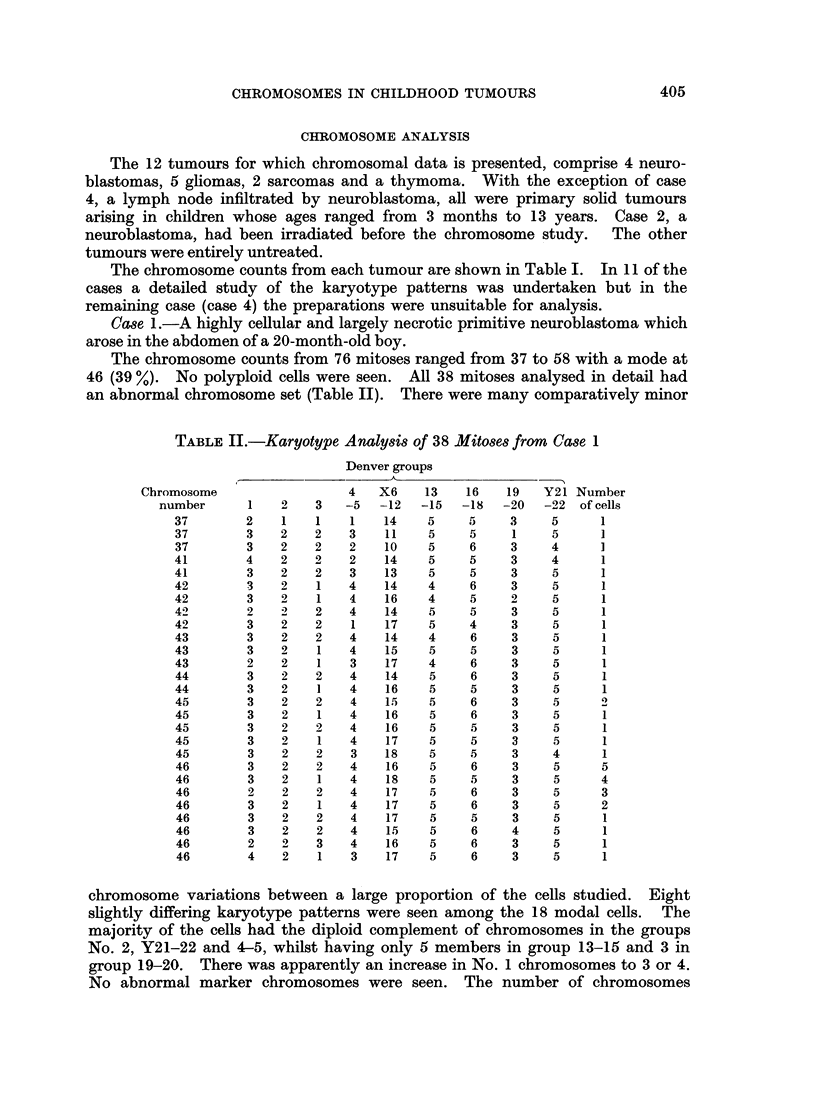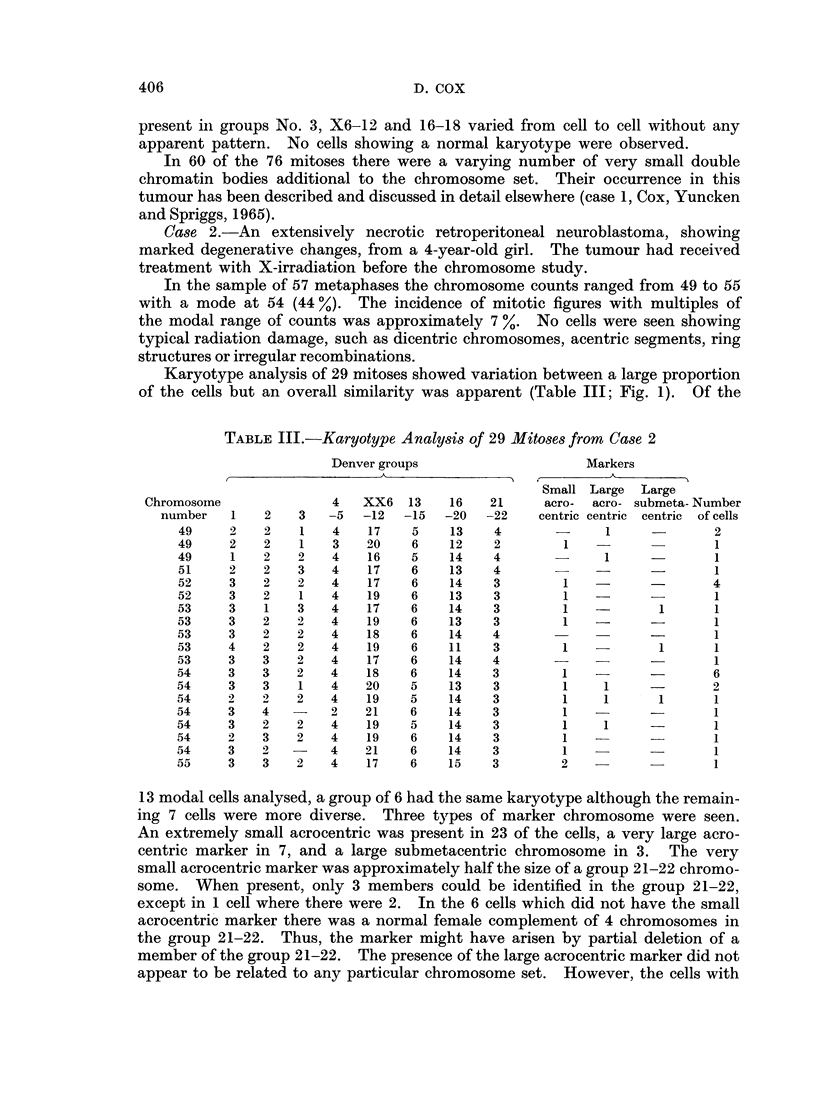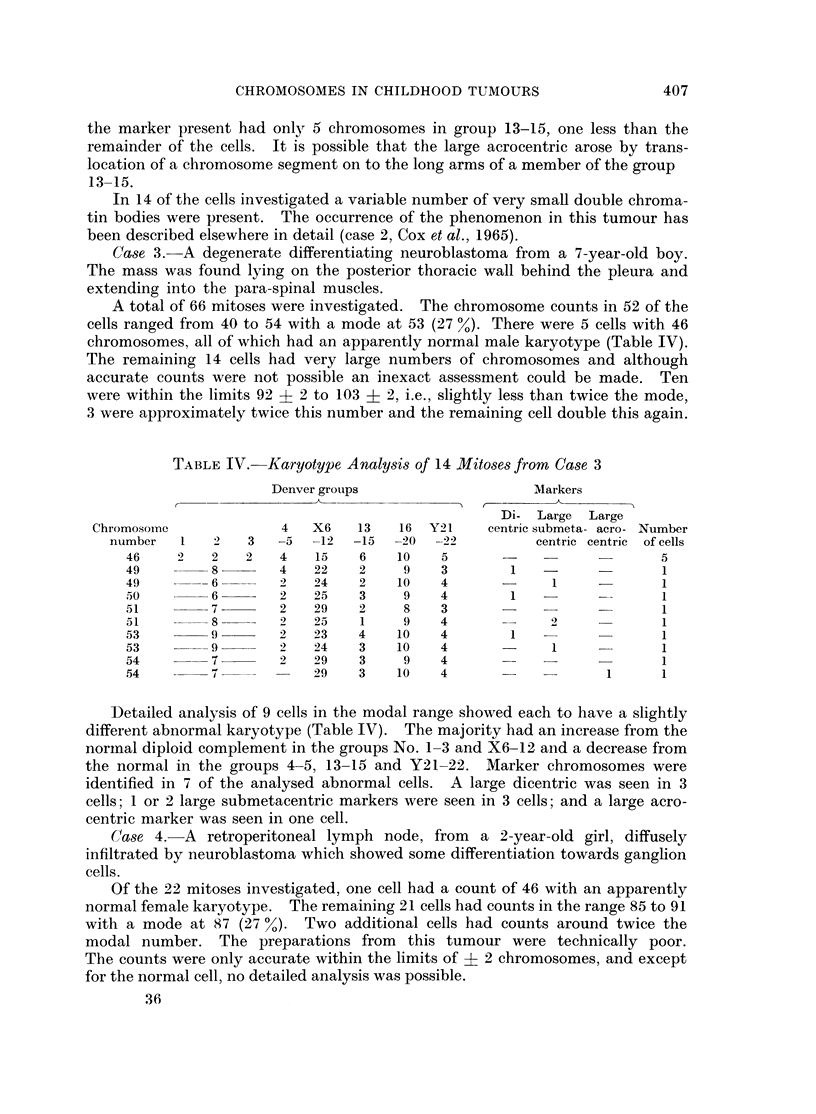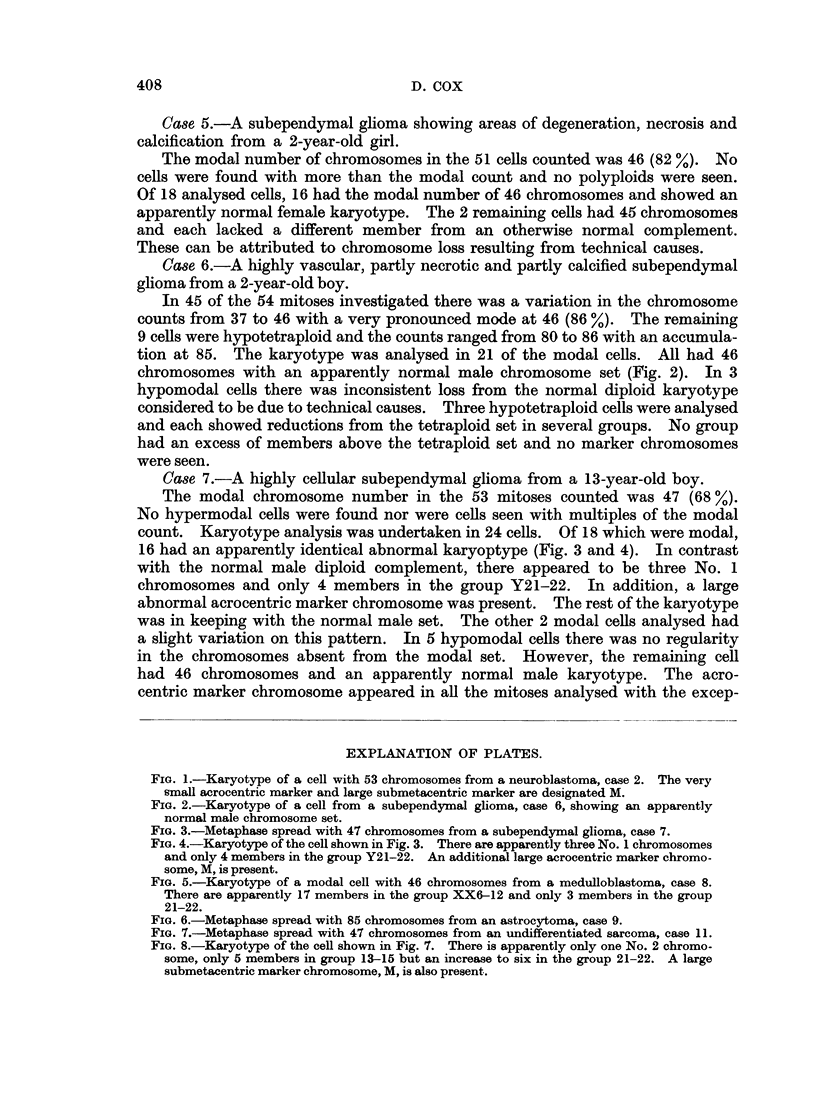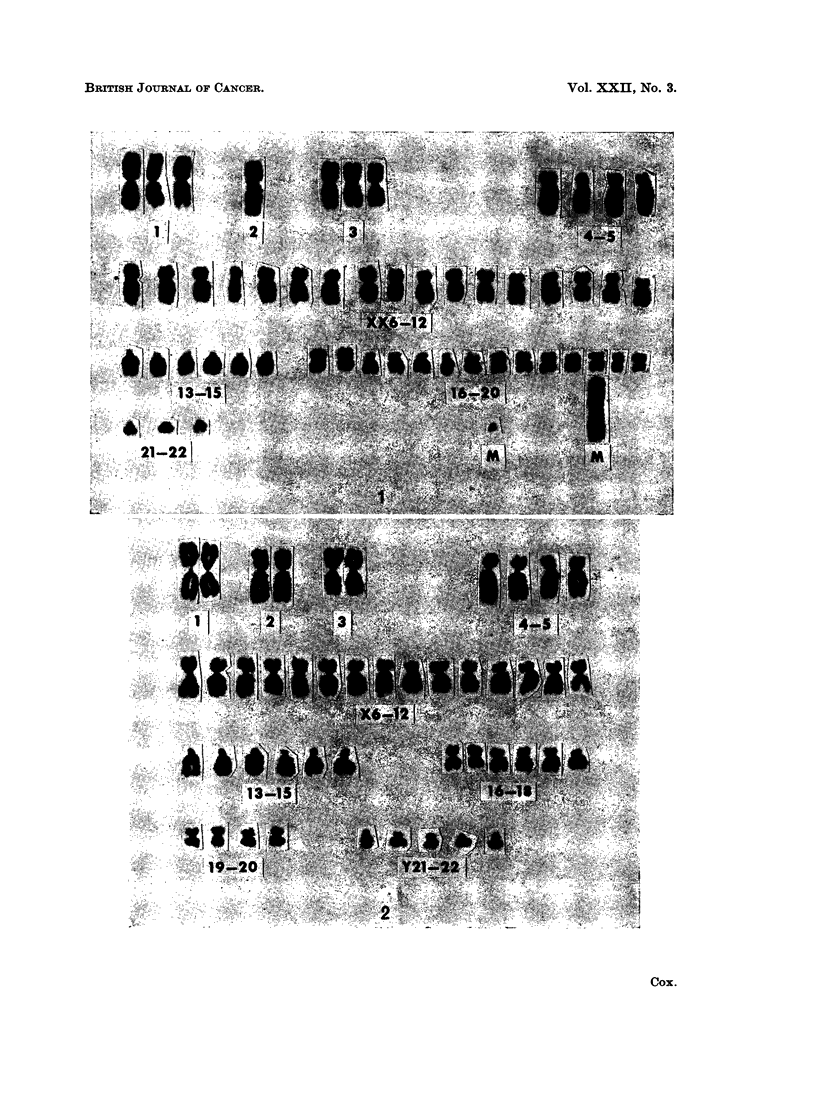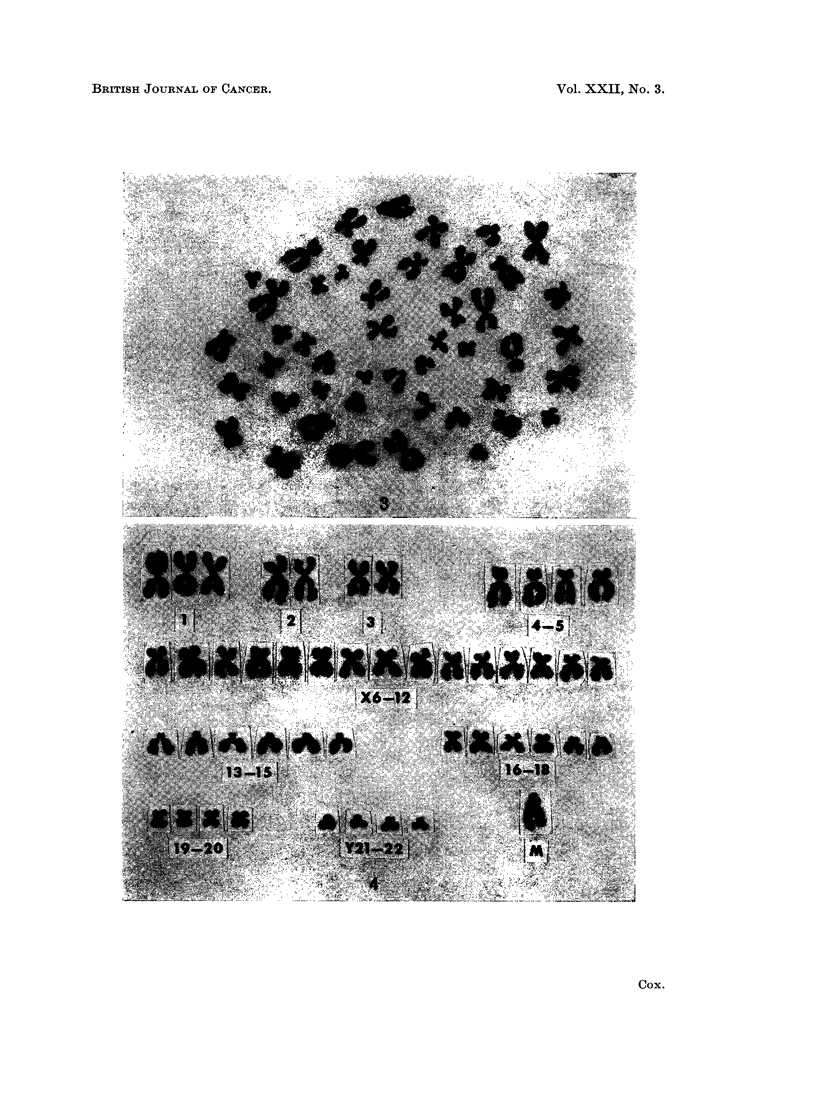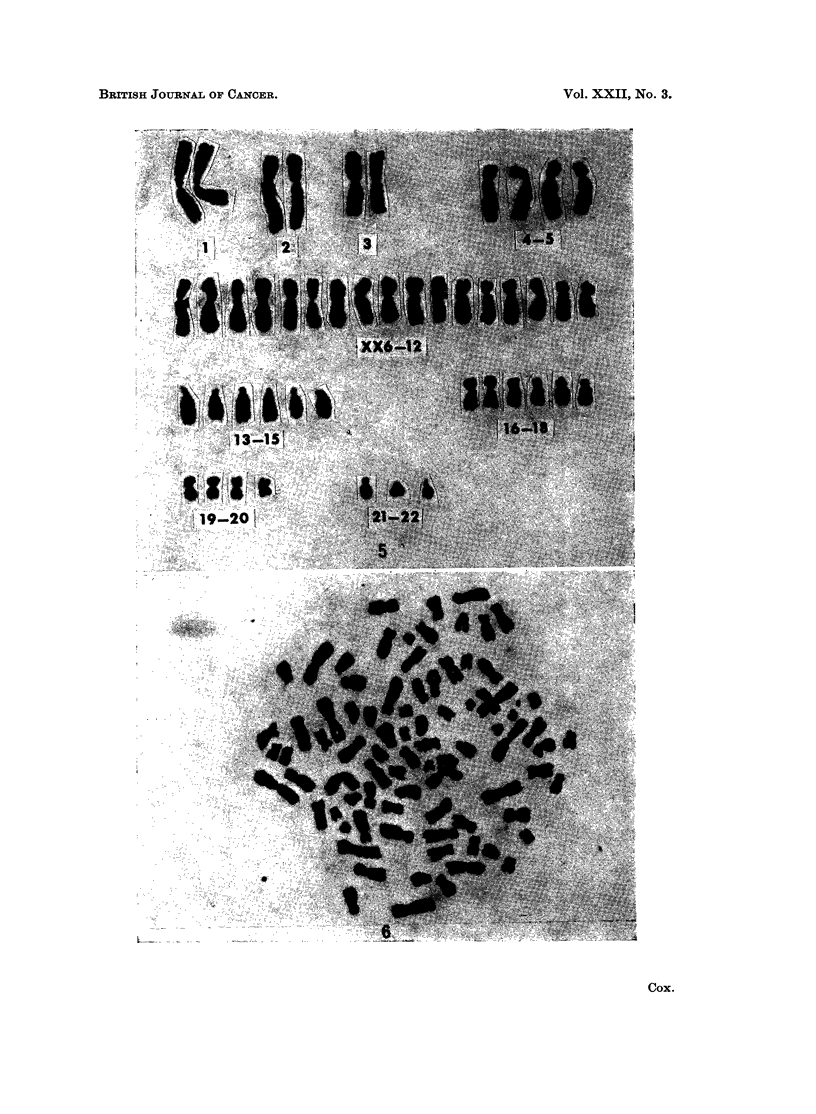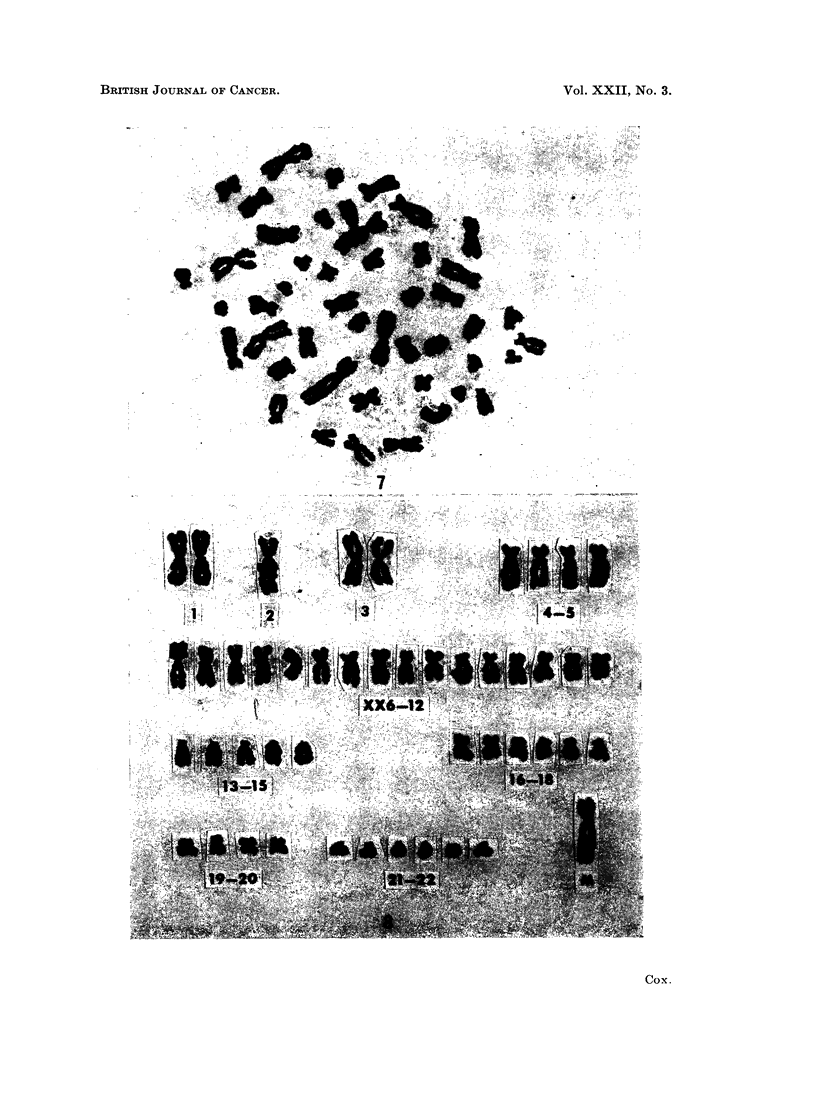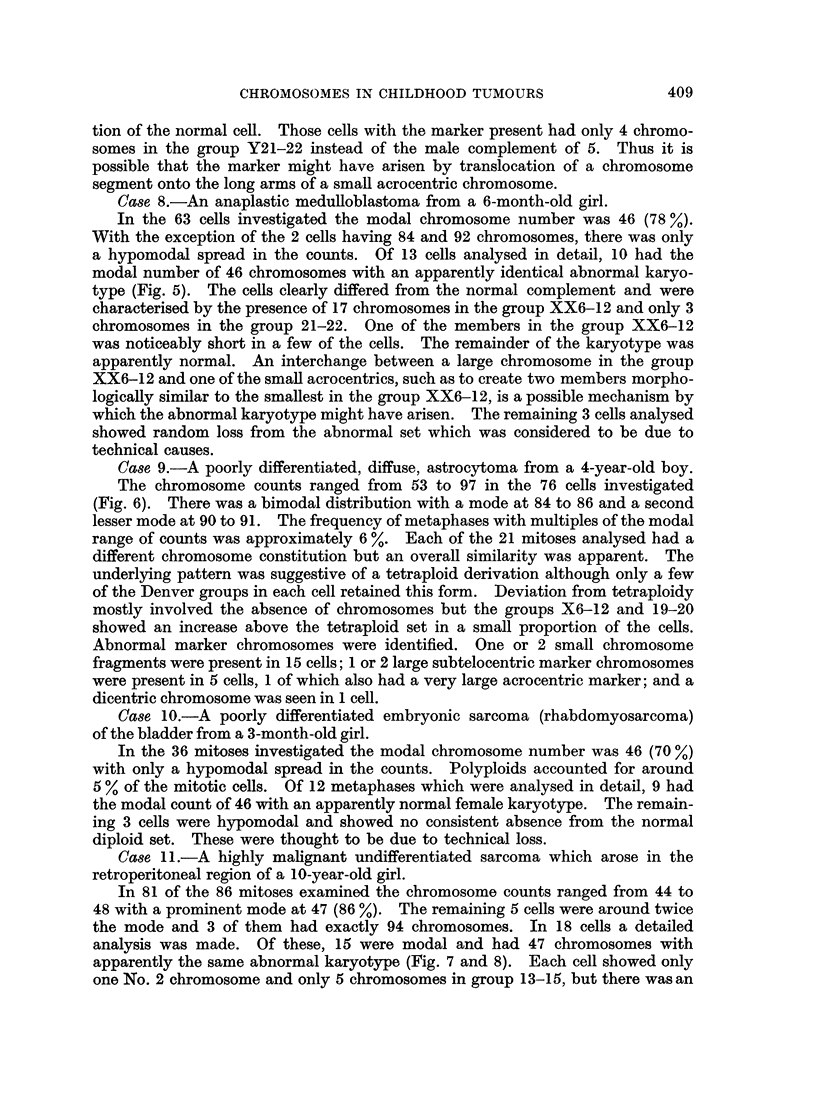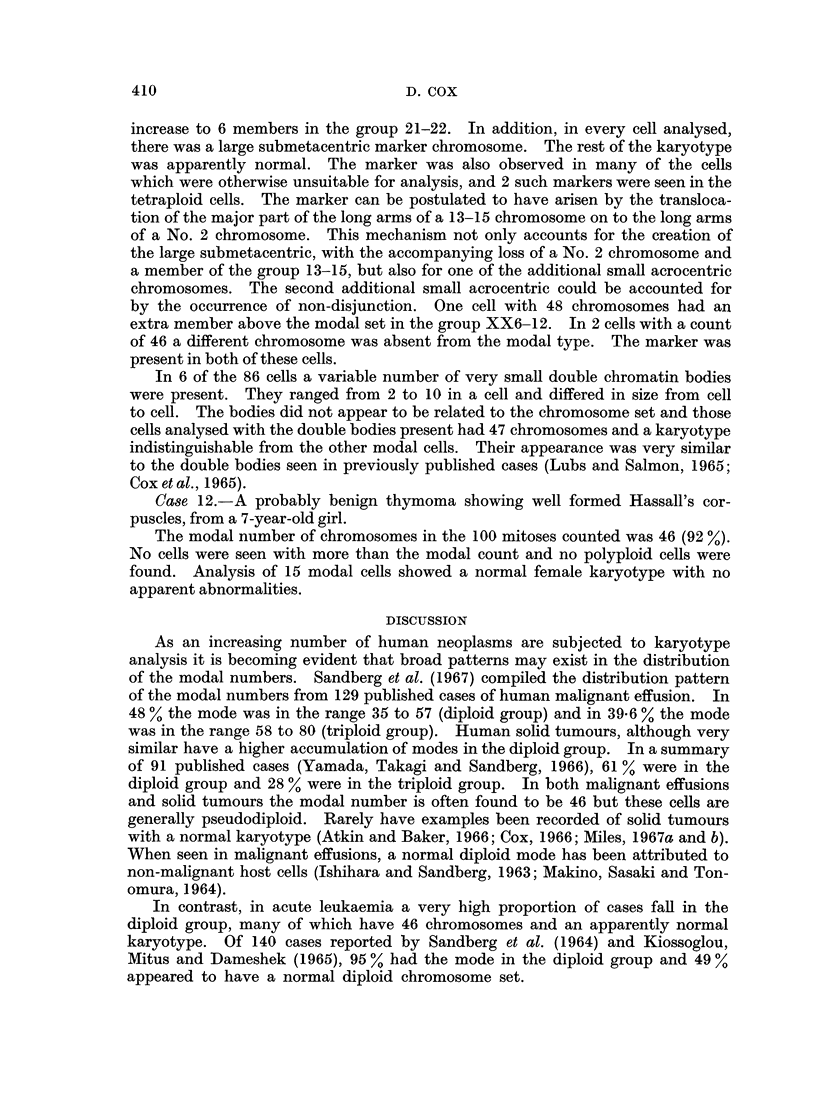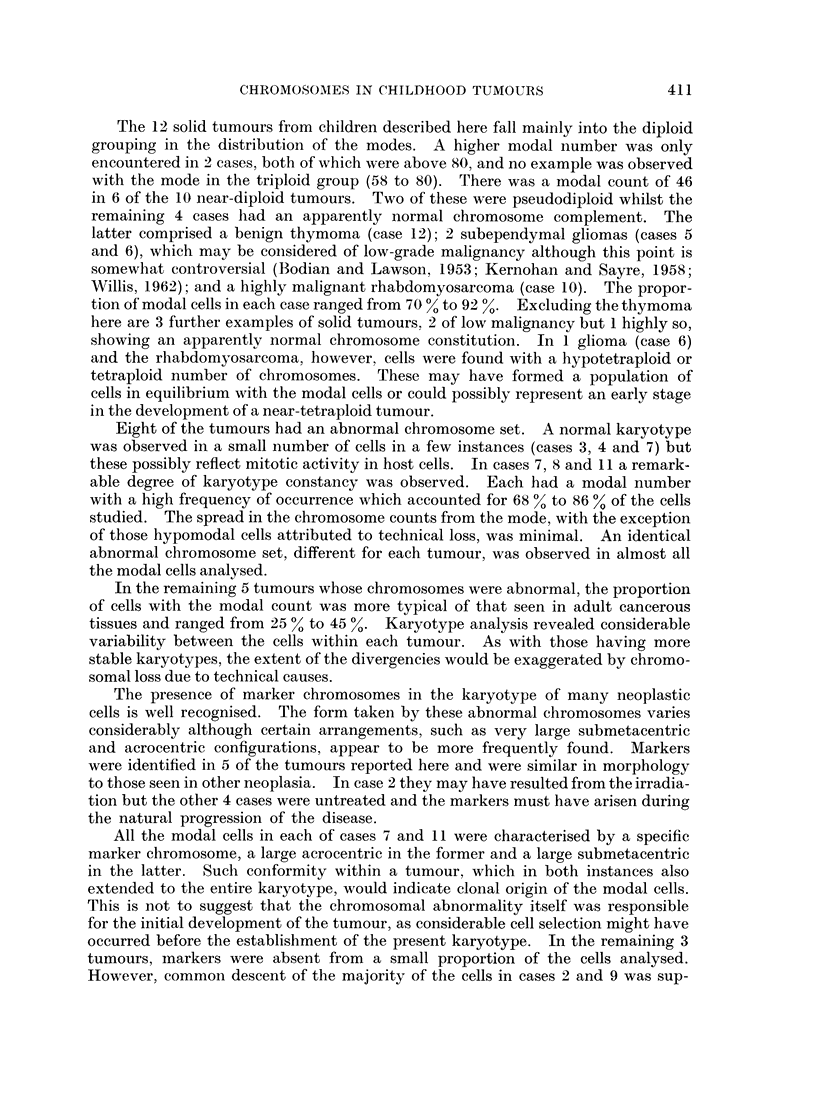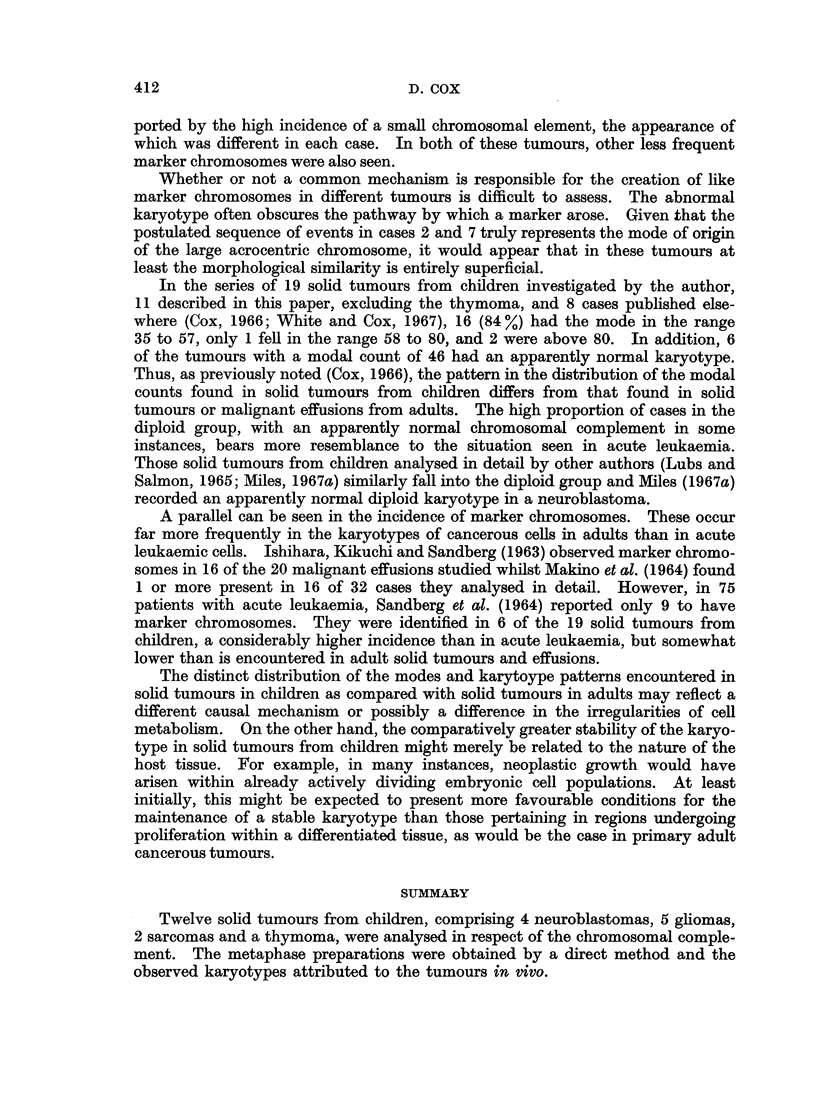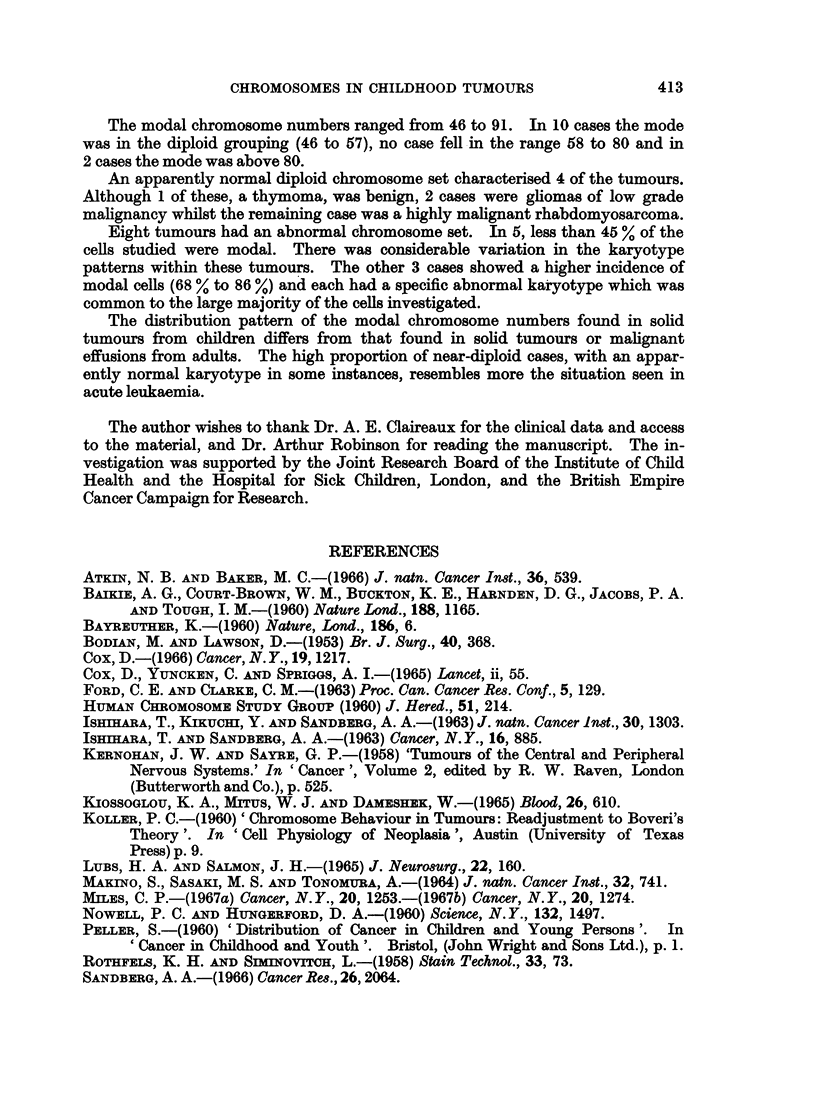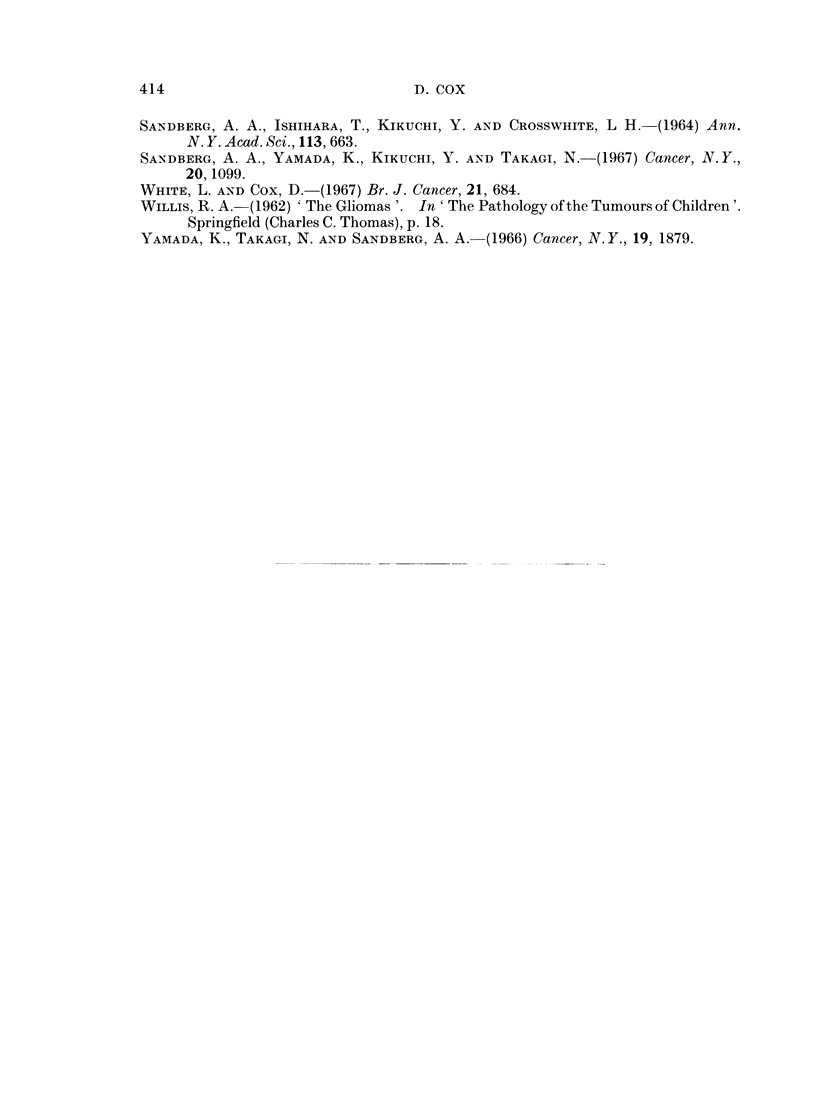# Chromosome studies in 12 solid tumours from children.

**DOI:** 10.1038/bjc.1968.48

**Published:** 1968-09

**Authors:** D. Cox

## Abstract

**Images:**


### CHROMOSOME STUDIES IN 12 SOLID TUMOURS FROM CHILDREN

D. COX

*From the *Department of Morbid Anatomy, Institute of Child Health and Hospital for Sick Children, Great Ormond Street, London. W.C.1.*

Received for publication March 25, 1968

IT is a widely accepted opinion that the gross chromosomal abnormalities observed in human cancer and leukaemia, with the possible exception of chronic myeloid leukaemia, are not involved in the immediate causation of neoplastic behaviour. The abnormalities probably arise as a secondary phenomenon, following the process of malignant transformation, and may well result from metabolic disturbances produced by the uncontrolled proliferation and abnormal growth (Bayreuther, 1960; Koller, 1960; Sandberg, 1966). However, common karyological features have been found to characterise the cells within many tumours. In a number of cases there is a closely similar or even an identical chromosome complement in a large proportion of the cells investigated.

Ford and Clarke (1963) presented a detailed argument demonstrating that clonal proliferation had occurred in three primary reticular neoplasms from man and the Chinese hamster. The opinion was advanced that clonal derivation would apply to all neoplasms possessing karyotypes that differ from the normal by more than one step. Further, it was concluded that the changes are of direct selective significance. This being so, alteration of the chromosomal complement during neoplastic growth cannot be entirely random and thus the karyotype may well play an important role in the course of the disease. For example, in the case of chronic myeloid leukaemia cited by Ford and Clarke (1963), alteration of the chromosome pattern in the blood and bone marrow accompanied the appearance of blast cells in the patient's peripheral blood.

In order to establish the spectrum of chromosomal abnormalities which may be encountered in human neoplasms, a large number of solid tumours, malignant effusions and leukaemias have been studied by many workers. With the exception of chronic myeloid leukaemia, characterised by the Philadelphia chromosome (Nowell and Hungerford, 1960; Baikie *et al.*, 1960), and those leukaemias and related disorders with apparently normal chromosomes, each case has been found to have a unique pattern of abnormal karyotypes which differ from that of any other case. Even within the cells of a single tumour considerable variation in the chromosomes may be encountered. There appears to be little correlation between the chromosomal complement and the histological description or degree of malignancy.

Detailed chromosomal analysis of solid tumours from children has only been recorded for a small number of cases. This must be largely due to their rare occurrence. Of all cancers, 1·5 to 1·8% occur during the first 15 years of life

* Present address: Department of Biophysics, University of Colorado Medical Center, 4200 East Ninth Avenue, Denver, Colorado 80220, U.S.A.

and of these around 45 % are leukaemia (Peller, 1960). In the present report the chromosomal complement in 12 solid tumours arising in children is documented and some comparisons are drawn between the karyotypes in these and other types of neoplasia.

### METHOD

The chromosome preparations were made directly, relying on those cells entering mitosis at the time of surgical operation. The method was a modification of the air drying technique of Rothfels and Siminovitch (1958).

A small piece of tumour was taken into 5 ml. of tissue culture medium TC 199 containing 5 μg./ml. of Colcemid (Ciba Ltd.). The Colcemid was allowed to act for 1 hour during which time the tissue was cut up with scissors to obtain as fine a suspension of cells as possible. Any pieces which could not be dispersed were removed and discarded. The suspension was then centrifuged at 1000 r.p.m. for 10 minutes, the supernatant fluid discarded, and the cells resuspended for 15 minutes in 5 ml. of 0·95 % sodium citrate solution prewarmed to 37° C. After hypotonic treatment, the suspension was centrifuged at 1000 r.p.m. for 10 minutes and all but 0·2 ml. of the supernatant solution was discarded. The pellet of cells was thoroughly dispersed in the remaining small quantity of fluid by being drawn in and out of a fine pipette. Freshly prepared, ice-cooled fixative, consisting of one part glacial acetic acid and three parts methanol, was then slowly added to the cells in a dropwise fashion down the side of the tube. The suspension was shaken after each addition of a few drops. When approximately 4 ml. of fixative had been added the cells were immediately spun down at 1000 r.p.m. for 10 minutes and the supernatant fixative discarded. The cell button was resuspended in fresh fixative and set aside for at least 1 hour at 0°C.

Just before the spreading of the chromosomes, the cells were transferred into 2 to 5 ml. of 45 % acetic acid. A film of condensation was formed on the surface of a clean slide by cooling on ice after which 4 or 5 drops of the cell suspension in 45 % acetic acid were pipetted onto it. The slide was gently heated over a low Bunsen flame until it was dry. The preparations were stained with 1 % acetic orcein and mounted permanently.

Processing was carried out immediately the specimens were received in the laboratory. No culture procedures were used to obtain mitotic enhancement so the possibility of *in vitro* cell selection or chromosomal change can be eliminated and the observed karyotypes directly attributed to the tumour *in vivo*.

The chromosome number was recorded for those cells where a reliable count could be made (tumour 4 excepted) and karyotype analysis, with the aid of photographs, was undertaken in all cells of sufficient clarity. The chromosomes were classified in terms of the Denver system of nomenclature of human mitotic chromosomes (Human Chromosome Study Group, 1960), whilst those chromosomes whose morphology was unlike that of any typical human chromosome were set aside as markers. In a few of the tumours, the members of some of the Denver groups tended to show slightly wider limits of overall length compared with the normal karyotype. This was particularly so for the group X6-12. However, in the analysis they were not considered to be sufficiently irregular to permit their separate classification. It must be emphasised that on allocation of a chromosome to a particular Denver group from morphological considerations it cannot be inferred that genetic homology exists with that group in the normal karyotype.

TABLE I.—*Chromosome Counts from 12 Solid Tumours from Children*

| Case No. | Tumour type | Chromosome Number <40 | 41 | 42 | 43 | 44 | 45 | 46 | 47 | 48 | 49 | 50 | 51 | 52 | 53 | 54 | 55 |
|---|---|---|---|---|---|---|---|---|---|---|---|---|---|---|---|---|---|
| 1 | Neuroblastoma . . . . . . . | 10 | 5 | 6 | 4 | 8 | 10 | 30 | 2 | — | — | — | — | — | — | — | — |
| 2 | Neuroblastoma . . . . . . . | — | — | — | — | — | — | — | — | — | 4 | — | 5 | 8 | 12 | 25 | 3 |
| 3 | Neuroblastoma . . . . . . . | 1 | — | — | — | — | — | 5 | — | — | 3 | 3 | 5 | 9 | 14 | 12 | — |
| 4 | Neuroblastoma . . . . . . . | — | — | — | — | — | — | 1 | — | — | — | — | — | — | — | — | — |
| 5 | Subependymal glioma . . . . . | 2 | — | — | 2 | 1 | 4 | 42 | — | — | — | — | — | — | — | — | — |
| 6 | Subependymal glioma . . . . . | 1 | 1 | — | 2 | 1 | 1 | 39 | — | — | — | — | — | — | — | — | — |
| 7 | Subependymal glioma . . . . . | 2 | 1 | 1 | 2 | 1 | 2 | 8 | 36 | — | — | — | — | — | — | — | — |
| 8 | Medulloblastoma . . . . . . | 3 | — | — | 2 | 4 | 4 | 48 | — | — | — | — | — | — | — | — | — |
| 9 | Astrocytoma . . . . . . . | — | — | — | — | — | — | — | — | — | — | — | — | — | 1 | — | — |
| 10 | Rhabdomyosarcoma . . . . . . | 6 | — | 1 | 2 | — | 2 | 25 | — | — | — | — | — | — | — | — | — |
| 11 | Undifferentiated sarcoma . . . . | — | — | — | — | 1 | 1 | 6 | 70 | 3 | — | — | — | — | — | — | — |
| 12 | Thymoma . . . . . . . | — | — | — | — | 2 | 6 | 92 | — | — | — | — | — | — | — | — | — |

| Case No. | Chromosome Number 56–79 | 80 | 81 | 82 | 83 | 84 | 85 | 86 | 87 | 88 | 89 | 90 | 91 | 92 | 93 | 94 | 95 | 96 | 97 | Total counts | %Modal multiples |
|---|---|---|---|---|---|---|---|---|---|---|---|---|---|---|---|---|---|---|---|---|---|
| 1 | 1 | — | — | — | — | — | — | — | — | — | — | — | — | — | — | — | — | — | — | 76 | — |
| 2 | — | — | — | — | — | — | — | — | — | — | — | — | — | — | — | — | — | — | — | 57 | 7 |
| 3 | — | — | — | — | — | — | — | — | — | — | — | — | — | — | — | — | — | — | — | 52 | 22 |
| 4 | — | — | — | — | — | — | 3 | 4 | 6 | 4 | 3 | — | 1 | — | — | — | — | — | — | 22 | 8 |
| 5 | — | — | — | — | — | — | — | — | — | — | — | — | — | — | — | — | — | — | — | 51 | — |
| 6 | — | 2 | — | — | — | 1 | 4 | 2 | — | — | — | — | — | — | — | — | — | — | — | 54 | 17 |
| 7 | — | — | — | — | — | — | — | — | — | — | — | — | — | — | — | — | — | — | — | 53 | — |
| 8 | — | — | — | — | — | 1 | — | — | — | — | — | — | — | 1 | — | — | — | — | — | 63 | 2 |
| 9 | 6 | 1 | — | 2 | 4 | 11 | 10 | 13 | 5 | 1 | 3 | 6 | 6 | 3 | 3 | — | — | — | 1 | 76 | 6 |
| 10 | — | — | — | — | — | — | — | — | — | — | — | — | — | — | — | — | — | — | — | 36 | 5 |
| 11 | — | — | — | — | — | — | — | — | — | — | — | 1 | 1 | — | — | 3 | — | — | — | 86 | 6 |
| 12 | — | — | — | — | — | — | — | — | — | — | — | — | — | — | — | — | — | — | — | 100 | — |

### CHROMOSOME ANALYSIS

The 12 tumours for which chromosomal data is presented, comprise 4 neuroblastomas, 5 gliomas, 2 sarcomas and a thymoma. With the exception of case 4, a lymph node infiltrated by neuroblastoma, all were primary solid tumours arising in children whose ages ranged from 3 months to 13 years. Case 2, a neuroblastoma, had been irradiated before the chromosome study. The other tumours were entirely untreated.

The chromosome counts from each tumour are shown in Table I. In 11 of the cases a detailed study of the karyotype patterns was undertaken but in the remaining case (case 4) the preparations were unsuitable for analysis.

*Case* 1.—A highly cellular and largely necrotic primitive neuroblastoma which arose in the abdomen of a 20-month-old boy.

The chromosome counts from 76 mitoses ranged from 37 to 58 with a mode at 46 (39 %). No polyploid cells were seen. All 38 mitoses analysed in detail had an abnormal chromosome set (Table II). There were many comparatively minor

TABLE II.—*Karyotype Analysis of* 38 *Mitoses from Case* 1

| Chromosome number | Denver groups 1 | 2 | 3 | 4–5 | X6–12 | 13–15 | 16–18 | 19–20 | Y21–22 | Number of cells |
|---|---|---|---|---|---|---|---|---|---|---|
| 37 | 2 | 1 | 1 | 1 | 14 | 5 | 5 | 3 | 5 | 1 |
| 37 | 3 | 2 | 2 | 3 | 11 | 5 | 5 | 1 | 5 | 1 |
| 37 | 3 | 2 | 2 | 2 | 10 | 5 | 6 | 3 | 4 | 1 |
| 41 | 4 | 2 | 2 | 2 | 14 | 5 | 5 | 3 | 4 | 1 |
| 41 | 3 | 2 | 2 | 3 | 13 | 5 | 5 | 3 | 5 | 1 |
| 42 | 3 | 2 | 1 | 4 | 14 | 4 | 6 | 3 | 5 | 1 |
| 42 | 3 | 2 | 1 | 4 | 16 | 4 | 5 | 2 | 5 | 1 |
| 42 | 2 | 2 | 2 | 4 | 14 | 5 | 5 | 3 | 5 | 1 |
| 42 | 3 | 2 | 2 | 1 | 17 | 5 | 4 | 3 | 5 | 1 |
| 43 | 3 | 2 | 2 | 4 | 14 | 4 | 6 | 3 | 5 | 1 |
| 43 | 3 | 2 | 1 | 4 | 15 | 5 | 5 | 3 | 5 | 1 |
| 43 | 2 | 2 | 1 | 3 | 17 | 4 | 6 | 3 | 5 | 1 |
| 44 | 3 | 2 | 2 | 4 | 14 | 5 | 6 | 3 | 5 | 1 |
| 44 | 3 | 2 | 1 | 4 | 16 | 5 | 5 | 3 | 5 | 1 |
| 45 | 3 | 2 | 2 | 4 | 15 | 5 | 6 | 3 | 5 | 2 |
| 45 | 3 | 2 | 1 | 4 | 16 | 5 | 6 | 3 | 5 | 1 |
| 45 | 3 | 2 | 2 | 4 | 16 | 5 | 5 | 3 | 5 | 1 |
| 45 | 3 | 2 | 1 | 4 | 17 | 5 | 5 | 3 | 5 | 1 |
| 45 | 3 | 2 | 2 | 3 | 18 | 5 | 5 | 3 | 4 | 1 |
| 46 | 3 | 2 | 2 | 4 | 16 | 5 | 6 | 3 | 5 | 5 |
| 46 | 3 | 2 | 1 | 4 | 18 | 5 | 5 | 3 | 5 | 4 |
| 46 | 2 | 2 | 2 | 4 | 17 | 5 | 6 | 3 | 5 | 3 |
| 46 | 3 | 2 | 1 | 4 | 17 | 5 | 6 | 3 | 5 | 2 |
| 46 | 3 | 2 | 2 | 4 | 17 | 5 | 5 | 3 | 5 | 1 |
| 46 | 3 | 2 | 2 | 4 | 15 | 5 | 6 | 4 | 5 | 1 |
| 46 | 2 | 2 | 3 | 4 | 16 | 5 | 6 | 3 | 5 | 1 |
| 46 | 4 | 2 | 1 | 3 | 17 | 5 | 6 | 3 | 5 | 1 |

chromosome variations between a large proportion of the cells studied. Eight slightly differing karyotype patterns were seen among the 18 modal cells. The majority of the cells had the diploid complement of chromosomes in the groups No. 2, Y21–22 and 4–5, whilst having only 5 members in group 13–15 and 3 in group 19–20. There was apparently an increase in No. 1 chromosomes to 3 or 4. No abnormal marker chromosomes were seen. The number of chromosomes present in groups No. 3, X6–12 and 16–18 varied from cell to cell without any apparent pattern. No cells showing a normal karyotype were observed.

In 60 of the 76 mitoses there were a varying number of very small double chromatin bodies additional to the chromosome set. Their occurrence in this tumour has been described and discussed in detail elsewhere (case 1, Cox, Yuncken and Spriggs, 1965).

*Case* 2.—An extensively necrotic retroperitoneal neuroblastoma, showing marked degenerative changes, from a 4-year-old girl. The tumour had received treatment with X-irradiation before the chromosome study.

In the sample of 57 metaphases the chromosome counts ranged from 49 to 55 with a mode at 54 (44 %). The incidence of mitotic figures with multiples of the modal range of counts was approximately 7 %. No cells were seen showing typical radiation damage, such as dicentric chromosomes, acentric segments, ring structures or irregular recombinations.

Karyotype analysis of 29 mitoses showed variation between a large proportion of the cells but an overall similarity was apparent (Table III; Fig. 1). Of the

TABLE III.—*Karyotype Analysis of* 29 *Mitoses from Case* 2

| | Denver groups | | | | | | | | Markers | | | |
|---|---|---|---|---|---|---|---|---|---|---|---|---|
| Chromosome number | 1 | 2 | 3 | 4–5 | XX6–12 | 13–15 | 16–20 | 21–22 | Small acrocentric | Large acrocentric | Large submetacentric | Number of cells |
| 49 | 2 | 2 | 1 | 4 | 17 | 5 | 13 | 4 | — | 1 | — | 2 |
| 49 | 2 | 2 | 1 | 3 | 20 | 6 | 12 | 2 | 1 | — | — | 1 |
| 49 | 1 | 2 | 2 | 4 | 16 | 5 | 14 | 4 | — | 1 | — | 1 |
| 51 | 2 | 2 | 3 | 4 | 17 | 6 | 13 | 4 | — | — | — | 1 |
| 52 | 3 | 2 | 2 | 4 | 17 | 6 | 14 | 3 | 1 | — | — | 4 |
| 52 | 3 | 2 | 1 | 4 | 19 | 6 | 13 | 3 | 1 | — | — | 1 |
| 53 | 3 | 1 | 3 | 4 | 17 | 6 | 14 | 3 | 1 | — | 1 | 1 |
| 53 | 3 | 2 | 2 | 4 | 19 | 6 | 13 | 3 | 1 | — | — | 1 |
| 53 | 3 | 2 | 2 | 4 | 18 | 6 | 14 | 4 | — | — | — | 1 |
| 53 | 4 | 2 | 2 | 4 | 19 | 6 | 11 | 3 | 1 | — | 1 | 1 |
| 53 | 3 | 3 | 2 | 4 | 17 | 6 | 14 | 4 | — | — | — | 1 |
| 54 | 3 | 3 | 2 | 4 | 18 | 6 | 14 | 3 | 1 | — | — | 6 |
| 54 | 3 | 3 | 1 | 4 | 20 | 5 | 13 | 3 | 1 | 1 | — | 2 |
| 54 | 2 | 2 | 2 | 4 | 19 | 5 | 14 | 3 | 1 | 1 | 1 | 1 |
| 54 | 3 | 4 | — | 2 | 21 | 6 | 14 | 3 | 1 | — | — | 1 |
| 54 | 3 | 2 | 2 | 4 | 19 | 5 | 14 | 3 | 1 | 1 | — | 1 |
| 54 | 2 | 3 | 2 | 4 | 19 | 6 | 14 | 3 | 1 | — | — | 1 |
| 54 | 3 | 2 | — | 4 | 21 | 6 | 14 | 3 | 1 | — | — | 1 |
| 55 | 3 | 3 | 2 | 4 | 17 | 6 | 15 | 3 | 2 | — | — | 1 |

13 modal cells analysed, a group of 6 had the same karyotype although the remaining 7 cells were more diverse. Three types of marker chromosome were seen. An extremely small acrocentric was present in 23 of the cells, a very large acrocentric marker in 7, and a large submetacentric chromosome in 3. The very small acrocentric marker was approximately half the size of a group 21–22 chromosome. When present, only 3 members could be identified in the group 21–22, except in 1 cell where there were 2. In the 6 cells which did not have the small acrocentric marker there was a normal female complement of 4 chromosomes in the group 21–22. Thus, the marker might have arisen by partial deletion of a member of the group 21–22. The presence of the large acrocentric marker did not appear to be related to any particular chromosome set. However, the cells with the marker present had only 5 chromosomes in group 13–15, one less than the remainder of the cells. It is possible that the large acrocentric arose by translocation of a chromosome segment on to the long arms of a member of the group 13–15.

In 14 of the cells investigated a variable number of very small double chromatin bodies were present. The occurrence of the phenomenon in this tumour has been described elsewhere in detail (case 2, Cox *et al.*, 1965).

*Case* 3.—A degenerate differentiating neuroblastoma from a 7-year-old boy. The mass was found lying on the posterior thoracic wall behind the pleura and extending into the para-spinal muscles.

A total of 66 mitoses were investigated. The chromosome counts in 52 of the cells ranged from 40 to 54 with a mode at 53 (27 %). There were 5 cells with 46 chromosomes, all of which had an apparently normal male karyotype (Table IV). The remaining 14 cells had very large numbers of chromosomes and although accurate counts were not possible an inexact assessment could be made. Ten were within the limits 92 $\pm$ 2 to 103 $\pm$ 2, i.e., slightly less than twice the mode, 3 were approximately twice this number and the remaining cell double this again.

TABLE IV.—*Karyotype Analysis of* 14 *Mitoses from Case* 3

| Chromosome number | Denver groups: 1 | 2 | 3 | 4–5 | X6–12 | 13–15 | 16–20 | Y21–22 | Markers: Dicentric | Large submetacentric | Large acrocentric | Number of cells |
|---|---|---|---|---|---|---|---|---|---|---|---|---|
| 46 | 2 | 2 | 2 | 4 | 15 | 6 | 10 | 5 | — | — | — | 5 |
| 49 | | ——8—— | | 4 | 22 | 2 | 9 | 3 | 1 | — | — | 1 |
| 49 | | ——6—— | | 2 | 24 | 2 | 10 | 4 | — | 1 | — | 1 |
| 50 | | ——6—— | | 2 | 25 | 3 | 9 | 4 | 1 | — | — | 1 |
| 51 | | ——7—— | | 2 | 29 | 2 | 8 | 3 | — | — | — | 1 |
| 51 | | ——8—— | | 2 | 25 | 1 | 9 | 4 | — | 2 | — | 1 |
| 53 | | ——9—— | | 2 | 23 | 4 | 10 | 4 | 1 | — | — | 1 |
| 53 | | ——9—— | | 2 | 24 | 3 | 10 | 4 | — | 1 | — | 1 |
| 54 | | ——7—— | | 2 | 29 | 3 | 9 | 4 | — | — | — | 1 |
| 54 | | ——7—— | | — | 29 | 3 | 10 | 4 | — | — | 1 | 1 |

Detailed analysis of 9 cells in the modal range showed each to have a slightly different abnormal karyotype (Table IV). The majority had an increase from the normal diploid complement in the groups No. 1–3 and X6–12 and a decrease from the normal in the groups 4–5, 13–15 and Y21–22. Marker chromosomes were identified in 7 of the analysed abnormal cells. A large dicentric was seen in 3 cells; 1 or 2 large submetacentric markers were seen in 3 cells; and a large acrocentric marker was seen in one cell.

*Case* 4.—A retroperitoneal lymph node, from a 2-year-old girl, diffusely infiltrated by neuroblastoma which showed some differentiation towards ganglion cells.

Of the 22 mitoses investigated, one cell had a count of 46 with an apparently normal female karyotype. The remaining 21 cells had counts in the range 85 to 91 with a mode at 87 (27 %). Two additional cells had counts around twice the modal number. The preparations from this tumour were technically poor. The counts were only accurate within the limits of $\pm$ 2 chromosomes, and except for the normal cell, no detailed analysis was possible.

*Case* 5.—A subependymal glioma showing areas of degeneration, necrosis and calcification from a 2-year-old girl.

The modal number of chromosomes in the 51 cells counted was 46 (82 %). No cells were found with more than the modal count and no polyploids were seen. Of 18 analysed cells, 16 had the modal number of 46 chromosomes and showed an apparently normal female karyotype. The 2 remaining cells had 45 chromosomes and each lacked a different member from an otherwise normal complement. These can be attributed to chromosome loss resulting from technical causes.

*Case* 6.—A highly vascular, partly necrotic and partly calcified subependymal glioma from a 2-year-old boy.

In 45 of the 54 mitoses investigated there was a variation in the chromosome counts from 37 to 46 with a very pronounced mode at 46 (86 %). The remaining 9 cells were hypotetraploid and the counts ranged from 80 to 86 with an accumulation at 85. The karyotype was analysed in 21 of the modal cells. All had 46 chromosomes with an apparently normal male chromosome set (Fig. 2). In 3 hypomodal cells there was inconsistent loss from the normal diploid karyotype considered to be due to technical causes. Three hypotetraploid cells were analysed and each showed reductions from the tetraploid set in several groups. No group had an excess of members above the tetraploid set and no marker chromosomes were seen.

*Case* 7.—A highly cellular subependymal glioma from a 13-year-old boy.

The modal chromosome number in the 53 mitoses counted was 47 (68 %). No hypermodal cells were found nor were cells seen with multiples of the modal count. Karyotype analysis was undertaken in 24 cells. Of 18 which were modal, 16 had an apparently identical abnormal karyoptype (Fig. 3 and 4). In contrast with the normal male diploid complement, there appeared to be three No. 1 chromosomes and only 4 members in the group Y21–22. In addition, a large abnormal acrocentric marker chromosome was present. The rest of the karyotype was in keeping with the normal male set. The other 2 modal cells analysed had a slight variation on this pattern. In 5 hypomodal cells there was no regularity in the chromosomes absent from the modal set. However, the remaining cell had 46 chromosomes and an apparently normal male karyotype. The acrocentric marker chromosome appeared in all the mitoses analysed with the excep-

---

### EXPLANATION OF PLATES.

FIG. 1.—Karyotype of a cell with 53 chromosomes from a neuroblastoma, case 2. The very small acrocentric marker and large submetacentric marker are designated M.

FIG. 2.—Karyotype of a cell from a subependymal glioma, case 6, showing an apparently normal male chromosome set.

FIG. 3.—Metaphase spread with 47 chromosomes from a subependymal glioma, case 7.

FIG. 4.—Karyotype of the cell shown in Fig. 3. There are apparently three No. 1 chromosomes and only 4 members in the group Y21–22. An additional large acrocentric marker chromosome, M, is present.

FIG. 5.—Karyotype of a modal cell with 46 chromosomes from a medulloblastoma, case 8. There are apparently 17 members in the group XX6–12 and only 3 members in the group 21–22.

FIG. 6.—Metaphase spread with 85 chromosomes from an astrocytoma, case 9.

FIG. 7.—Metaphase spread with 47 chromosomes from an undifferentiated sarcoma, case 11.

FIG. 8.—Karyotype of the cell shown in Fig. 7. There is apparently only one No. 2 chromosome, only 5 members in group 13–15 but an increase to six in the group 21–22. A large submetacentric marker chromosome, M, is also present.

1
2
3
4–5
XX6–12
13–15
16–20
21–22
M
M

1

1
2
3
4–5
X6–12
13–15
16–18
19–20
Y21–22

2

Cox.

3

4

Cox.

5

6

Cox.

7

8

Cox.

tion of the normal cell. Those cells with the marker present had only 4 chromosomes in the group Y21–22 instead of the male complement of 5. Thus it is possible that the marker might have arisen by translocation of a chromosome segment onto the long arms of a small acrocentric chromosome.

*Case* 8.—An anaplastic medulloblastoma from a 6-month-old girl.

In the 63 cells investigated the modal chromosome number was 46 (78 %). With the exception of the 2 cells having 84 and 92 chromosomes, there was only a hypomodal spread in the counts. Of 13 cells analysed in detail, 10 had the modal number of 46 chromosomes with an apparently identical abnormal karyotype (Fig. 5). The cells clearly differed from the normal complement and were characterised by the presence of 17 chromosomes in the group XX6–12 and only 3 chromosomes in the group 21–22. One of the members in the group XX6–12 was noticeably short in a few of the cells. The remainder of the karyotype was apparently normal. An interchange between a large chromosome in the group XX6–12 and one of the small acrocentrics, such as to create two members morphologically similar to the smallest in the group XX6–12, is a possible mechanism by which the abnormal karyotype might have arisen. The remaining 3 cells analysed showed random loss from the abnormal set which was considered to be due to technical causes.

*Case* 9.—A poorly differentiated, diffuse, astrocytoma from a 4-year-old boy.

The chromosome counts ranged from 53 to 97 in the 76 cells investigated (Fig. 6). There was a bimodal distribution with a mode at 84 to 86 and a second lesser mode at 90 to 91. The frequency of metaphases with multiples of the modal range of counts was approximately 6 %. Each of the 21 mitoses analysed had a different chromosome constitution but an overall similarity was apparent. The underlying pattern was suggestive of a tetraploid derivation although only a few of the Denver groups in each cell retained this form. Deviation from tetraploidy mostly involved the absence of chromosomes but the groups X6–12 and 19–20 showed an increase above the tetraploid set in a small proportion of the cells. Abnormal marker chromosomes were identified. One or 2 small chromosome fragments were present in 15 cells; 1 or 2 large subtelocentric marker chromosomes were present in 5 cells, 1 of which also had a very large acrocentric marker; and a dicentric chromosome was seen in 1 cell.

*Case* 10.—A poorly differentiated embryonic sarcoma (rhabdomyosarcoma) of the bladder from a 3-month-old girl.

In the 36 mitoses investigated the modal chromosome number was 46 (70 %) with only a hypomodal spread in the counts. Polyploids accounted for around 5 % of the mitotic cells. Of 12 metaphases which were analysed in detail, 9 had the modal count of 46 with an apparently normal female karyotype. The remaining 3 cells were hypomodal and showed no consistent absence from the normal diploid set. These were thought to be due to technical loss.

*Case* 11.—A highly malignant undifferentiated sarcoma which arose in the retroperitoneal region of a 10-year-old girl.

In 81 of the 86 mitoses examined the chromosome counts ranged from 44 to 48 with a prominent mode at 47 (86 %). The remaining 5 cells were around twice the mode and 3 of them had exactly 94 chromosomes. In 18 cells a detailed analysis was made. Of these, 15 were modal and had 47 chromosomes with apparently the same abnormal karyotype (Fig. 7 and 8). Each cell showed only one No. 2 chromosome and only 5 chromosomes in group 13–15, but there was an increase to 6 members in the group 21–22. In addition, in every cell analysed, there was a large submetacentric marker chromosome. The rest of the karyotype was apparently normal. The marker was also observed in many of the cells which were otherwise unsuitable for analysis, and 2 such markers were seen in the tetraploid cells. The marker can be postulated to have arisen by the translocation of the major part of the long arms of a 13–15 chromosome on to the long arms of a No. 2 chromosome. This mechanism not only accounts for the creation of the large submetacentric, with the accompanying loss of a No. 2 chromosome and a member of the group 13–15, but also for one of the additional small acrocentric chromosomes. The second additional small acrocentric could be accounted for by the occurrence of non-disjunction. One cell with 48 chromosomes had an extra member above the modal set in the group XX6–12. In 2 cells with a count of 46 a different chromosome was absent from the modal type. The marker was present in both of these cells.

In 6 of the 86 cells a variable number of very small double chromatin bodies were present. They ranged from 2 to 10 in a cell and differed in size from cell to cell. The bodies did not appear to be related to the chromosome set and those cells analysed with the double bodies present had 47 chromosomes and a karyotype indistinguishable from the other modal cells. Their appearance was very similar to the double bodies seen in previously published cases (Lubs and Salmon, 1965; Cox *et al.*, 1965).

*Case* 12.—A probably benign thymoma showing well formed Hassall's corpuscles, from a 7-year-old girl.

The modal number of chromosomes in the 100 mitoses counted was 46 (92 %). No cells were seen with more than the modal count and no polyploid cells were found. Analysis of 15 modal cells showed a normal female karyotype with no apparent abnormalities.

### DISCUSSION

As an increasing number of human neoplasms are subjected to karyotype analysis it is becoming evident that broad patterns may exist in the distribution of the modal numbers. Sandberg *et al.* (1967) compiled the distribution pattern of the modal numbers from 129 published cases of human malignant effusion. In 48 % the mode was in the range 35 to 57 (diploid group) and in 39·6 % the mode was in the range 58 to 80 (triploid group). Human solid tumours, although very similar have a higher accumulation of modes in the diploid group. In a summary of 91 published cases (Yamada, Takagi and Sandberg, 1966), 61 % were in the diploid group and 28 % were in the triploid group. In both malignant effusions and solid tumours the modal number is often found to be 46 but these cells are generally pseudodiploid. Rarely have examples been recorded of solid tumours with a normal karyotype (Atkin and Baker, 1966; Cox, 1966; Miles, 1967*a* and *b*). When seen in malignant effusions, a normal diploid mode has been attributed to non-malignant host cells (Ishihara and Sandberg, 1963; Makino, Sasaki and Tonomura, 1964).

In contrast, in acute leukaemia a very high proportion of cases fall in the diploid group, many of which have 46 chromosomes and an apparently normal karyotype. Of 140 cases reported by Sandberg *et al.* (1964) and Kiossoglou, Mitus and Dameshek (1965), 95 % had the mode in the diploid group and 49 % appeared to have a normal diploid chromosome set.

The 12 solid tumours from children described here fall mainly into the diploid grouping in the distribution of the modes. A higher modal number was only encountered in 2 cases, both of which were above 80, and no example was observed with the mode in the triploid group (58 to 80). There was a modal count of 46 in 6 of the 10 near-diploid tumours. Two of these were pseudodiploid whilst the remaining 4 cases had an apparently normal chromosome complement. The latter comprised a benign thymoma (case 12); 2 subependymal gliomas (cases 5 and 6), which may be considered of low-grade malignancy although this point is somewhat controversial (Bodian and Lawson, 1953; Kernohan and Sayre, 1958; Willis, 1962); and a highly malignant rhabdomyosarcoma (case 10). The proportion of modal cells in each case ranged from 70 % to 92 %. Excluding the thymoma here are 3 further examples of solid tumours, 2 of low malignancy but 1 highly so, showing an apparently normal chromosome constitution. In 1 glioma (case 6) and the rhabdomyosarcoma, however, cells were found with a hypotetraploid or tetraploid number of chromosomes. These may have formed a population of cells in equilibrium with the modal cells or could possibly represent an early stage in the development of a near-tetraploid tumour.

Eight of the tumours had an abnormal chromosome set. A normal karyotype was observed in a small number of cells in a few instances (cases 3, 4 and 7) but these possibly reflect mitotic activity in host cells. In cases 7, 8 and 11 a remarkable degree of karyotype constancy was observed. Each had a modal number with a high frequency of occurrence which accounted for 68 % to 86 % of the cells studied. The spread in the chromosome counts from the mode, with the exception of those hypomodal cells attributed to technical loss, was minimal. An identical abnormal chromosome set, different for each tumour, was observed in almost all the modal cells analysed.

In the remaining 5 tumours whose chromosomes were abnormal, the proportion of cells with the modal count was more typical of that seen in adult cancerous tissues and ranged from 25 % to 45 %. Karyotype analysis revealed considerable variability between the cells within each tumour. As with those having more stable karyotypes, the extent of the divergencies would be exaggerated by chromosomal loss due to technical causes.

The presence of marker chromosomes in the karyotype of many neoplastic cells is well recognised. The form taken by these abnormal chromosomes varies considerably although certain arrangements, such as very large submetacentric and acrocentric configurations, appear to be more frequently found. Markers were identified in 5 of the tumours reported here and were similar in morphology to those seen in other neoplasia. In case 2 they may have resulted from the irradiation but the other 4 cases were untreated and the markers must have arisen during the natural progression of the disease.

All the modal cells in each of cases 7 and 11 were characterised by a specific marker chromosome, a large acrocentric in the former and a large submetacentric in the latter. Such conformity within a tumour, which in both instances also extended to the entire karyotype, would indicate clonal origin of the modal cells. This is not to suggest that the chromosomal abnormality itself was responsible for the initial development of the tumour, as considerable cell selection might have occurred before the establishment of the present karyotype. In the remaining 3 tumours, markers were absent from a small proportion of the cells analysed. However, common descent of the majority of the cells in cases 2 and 9 was supported by the high incidence of a small chromosomal element, the appearance of which was different in each case. In both of these tumours, other less frequent marker chromosomes were also seen.

Whether or not a common mechanism is responsible for the creation of like marker chromosomes in different tumours is difficult to assess. The abnormal karyotype often obscures the pathway by which a marker arose. Given that the postulated sequence of events in cases 2 and 7 truly represents the mode of origin of the large acrocentric chromosome, it would appear that in these tumours at least the morphological similarity is entirely superficial.

In the series of 19 solid tumours from children investigated by the author, 11 described in this paper, excluding the thymoma, and 8 cases published elsewhere (Cox, 1966; White and Cox, 1967), 16 (84%) had the mode in the range 35 to 57, only 1 fell in the range 58 to 80, and 2 were above 80. In addition, 6 of the tumours with a modal count of 46 had an apparently normal karyotype. Thus, as previously noted (Cox, 1966), the pattern in the distribution of the modal counts found in solid tumours from children differs from that found in solid tumours or malignant effusions from adults. The high proportion of cases in the diploid group, with an apparently normal chromosomal complement in some instances, bears more resemblance to the situation seen in acute leukaemia. Those solid tumours from children analysed in detail by other authors (Lubs and Salmon, 1965; Miles, 1967*a*) similarly fall into the diploid group and Miles (1967*a*) recorded an apparently normal diploid karyotype in a neuroblastoma.

A parallel can be seen in the incidence of marker chromosomes. These occur far more frequently in the karyotypes of cancerous cells in adults than in acute leukaemic cells. Ishihara, Kikuchi and Sandberg (1963) observed marker chromosomes in 16 of the 20 malignant effusions studied whilst Makino *et al.* (1964) found 1 or more present in 16 of 32 cases they analysed in detail. However, in 75 patients with acute leukaemia, Sandberg *et al.* (1964) reported only 9 to have marker chromosomes. They were identified in 6 of the 19 solid tumours from children, a considerably higher incidence than in acute leukaemia, but somewhat lower than is encountered in adult solid tumours and effusions.

The distinct distribution of the modes and karytoype patterns encountered in solid tumours in children as compared with solid tumours in adults may reflect a different causal mechanism or possibly a difference in the irregularities of cell metabolism. On the other hand, the comparatively greater stability of the karyotype in solid tumours from children might merely be related to the nature of the host tissue. For example, in many instances, neoplastic growth would have arisen within already actively dividing embryonic cell populations. At least initially, this might be expected to present more favourable conditions for the maintenance of a stable karyotype than those pertaining in regions undergoing proliferation within a differentiated tissue, as would be the case in primary adult cancerous tumours.

### SUMMARY

Twelve solid tumours from children, comprising 4 neuroblastomas, 5 gliomas, 2 sarcomas and a thymoma, were analysed in respect of the chromosomal complement. The metaphase preparations were obtained by a direct method and the observed karyotypes attributed to the tumours *in vivo.*

The modal chromosome numbers ranged from 46 to 91. In 10 cases the mode was in the diploid grouping (46 to 57), no case fell in the range 58 to 80 and in 2 cases the mode was above 80.

An apparently normal diploid chromosome set characterised 4 of the tumours. Although 1 of these, a thymoma, was benign, 2 cases were gliomas of low grade malignancy whilst the remaining case was a highly malignant rhabdomyosarcoma.

Eight tumours had an abnormal chromosome set. In 5, less than 45 % of the cells studied were modal. There was considerable variation in the karyotype patterns within these tumours. The other 3 cases showed a higher incidence of modal cells (68 % to 86 %) and each had a specific abnormal karyotype which was common to the large majority of the cells investigated.

The distribution pattern of the modal chromosome numbers found in solid tumours from children differs from that found in solid tumours or malignant effusions from adults. The high proportion of near-diploid cases, with an apparently normal karyotype in some instances, resembles more the situation seen in acute leukaemia.

The author wishes to thank Dr. A. E. Claireaux for the clinical data and access to the material, and Dr. Arthur Robinson for reading the manuscript. The investigation was supported by the Joint Research Board of the Institute of Child Health and the Hospital for Sick Children, London, and the British Empire Cancer Campaign for Research.

### REFERENCES

Atkin, N. B. and Baker, M. C.—(1966) *J. natn. Cancer Inst.*, **36**, 539.
Baikie, A. G., Court-Brown, W. M., Buckton, K. E., Harnden, D. G., Jacobs, P. A. and Tough, I. M.—(1960) *Nature Lond.*, **188**, 1165.
Bayreuther, K.—(1960) *Nature, Lond.*, **186**, 6.
Bodian, M. and Lawson, D.—(1953) *Br. J. Surg.*, **40**, 368.
Cox, D.—(1966) *Cancer, N.Y.*, **19**, 1217.
Cox, D., Yuncken, C. and Spriggs, A. I.—(1965) *Lancet*, ii, 55.
Ford, C. E. and Clarke, C. M.—(1963) *Proc. Can. Cancer Res. Conf.*, **5**, 129.
Human Chromosome Study Group (1960) *J. Hered.*, **51**, 214.
Ishihara, T., Kikuchi, Y. and Sandberg, A. A.—(1963) *J. natn. Cancer Inst.*, **30**, 1303.
Ishihara, T. and Sandberg, A. A.—(1963) *Cancer, N.Y.*, **16**, 885.
Kernohan, J. W. and Sayre, G. P.—(1958) 'Tumours of the Central and Peripheral Nervous Systems.' *In* 'Cancer', Volume 2, edited by R. W. Raven, London (Butterworth and Co.), p. 525.
Kiossoglou, K. A., Mitus, W. J. and Dameshek, W.—(1965) *Blood*, **26**, 610.
Koller, P. C.—(1960) 'Chromosome Behaviour in Tumours: Readjustment to Boveri's Theory'. *In* 'Cell Physiology of Neoplasia', Austin (University of Texas Press) p. 9.
Lubs, H. A. and Salmon, J. H.—(1965) *J. Neurosurg.*, **22**, 160.
Makino, S., Sasaki, M. S. and Tonomura, A.—(1964) *J. natn. Cancer Inst.*, **32**, 741.
Miles, C. P.—(1967*a*) *Cancer, N.Y.*, **20**, 1253.—(1967*b*) *Cancer, N.Y.*, **20**, 1274.
Nowell, P. C. and Hungerford, D. A.—(1960) *Science, N.Y.*, **132**, 1497.
Peller, S.—(1960) 'Distribution of Cancer in Children and Young Persons'. In 'Cancer in Childhood and Youth'. Bristol, (John Wright and Sons Ltd.), p. 1.
Rothfels, K. H. and Siminovitch, L.—(1958) *Stain Technol.*, **33**, 73.
Sandberg, A. A.—(1966) *Cancer Res.*, **26**, 2064.

SANDBERG, A. A., ISHIHARA, T., KIKUCHI, Y. AND CROSSWHITE, L H.—(1964) *Ann. N.Y. Acad. Sci.*, **113**, 663.
SANDBERG, A. A., YAMADA, K., KIKUCHI, Y. AND TAKAGI, N.—(1967) *Cancer, N.Y.*, **20**, 1099.
WHITE, L. AND COX, D.—(1967) *Br. J. Cancer*, **21**, 684.
WILLIS, R. A.—(1962) ' The Gliomas '. *In* ' The Pathology of the Tumours of Children '. Springfield (Charles C. Thomas), p. 18.
YAMADA, K., TAKAGI, N. AND SANDBERG, A. A.—(1966) *Cancer, N.Y.*, **19**, 1879.